# HPV11 Positive Endometrioid Carcinoma of the Endometrium with Signet-Ring Cells: Diagnostic Criteria and Review of the Literature

**DOI:** 10.1155/2014/380571

**Published:** 2014-04-07

**Authors:** Teresa Pusiol

**Affiliations:** Institute of Anatomic Pathology, Rovereto Hospital, Rovereto, 38060 Trento, Italy

## Abstract

The presence of signet-ring cells in an endometrial adenocarcinoma is extremely uncommon and it is always necessary to rule out a metastatic neoplasm. We report a FIGO grade 2 endometrial carcinoma with a signet-ring cell component found in the curettage performed to a 53-year-old woman. The neoplastic proliferation was also found in the endometrium of the radical hysterectomy with bilateral salpingo-oophorectomy and pelvic and para-aortic lymphadenectomy. The uterine neoplasm invaded less than one-half of the myometrium (FIGO stage I B). Alcian blue showed the presence of mucin in the signet-ring cells. The patient was alive and without evidence of recurrence 14 months after surgery. Polymerase chain reaction method from paraffin-embedded tissue revealed the presence of human papilloma virus type 11. We have discussed the differential diagnosis of this kind of neoplasm and we have reviewed the literature on signet-ring cell carcinoma of the endometrium.

## 1. Introduction

Signet-ring cell (SRC) carcinoma is defined as a tumor composed predominantly or exclusively of SRCs, characterized by a central, optically clear, globoid droplet of cytoplasmic mucin with an eccentrically placed nucleus. SRCs are generally rare in primary adenocarcinomas of the female genital tract.

To the best of our knowledge primary carcinoma of the endometrium with SRCs has only been observed in four previous cases [1–3] (Table 1). In fact primary pure endometrial SRCs adenocarcinomas of the genital tract are extremely rare, while more often they may be seen admixed with other more conventional types. In this paper we report a new case of endometrial adenocarcinoma (EA) with SRCs component. We have reviewed the literature in order to emphasize the histological criteria in the diagnosis of this very unusual malignancy.

## 2. Materials and Methods

A 53-year-old multiparous (gravida 4, para 4) woman was referred to the Department of Obstetrics and Gynecology for persistent abnormal vaginal bleeding of three-month duration. An endometrial curettage was performed. An extensive search for an extrapelvic primary cancer was undertaken, but abdominopelvic computed tomography (CT), mammography, cystoscopy, esophagogastroduodenoscopy, and colonoscopy revealed no evidence of malignancy. The patient underwent a radical hysterectomy with bilateral salpingo-oophorectomy and pelvic and para-aortic lymph node dissection.

The patient provided written informed consent to perform the study. Tissue specimens were fixed in 10% neutral-buffered formalin and were paraffin-embedded according to standard procedures. Three-micrometer sections of representative blocks were deparaffinized in xylene, rehydrated, and treated with 3% H_2_O_2_ in TBS for 5 minutes to block endogenous peroxidase activity. Antigen retrieval procedure was performed by microwave oven heating in citrate buffer (pH 6) for each antibody. Cells expressing estrogen receptor (ER) (clone 6F11; Novocastra, Leica Byosystems Newcastle Ltd., UK), progesterone receptor (PR) (clone PGR-312; Novocastra, Leica Byosystems Newcastle Ltd., UK), and Ki-67 (clone MM1; Novocastra, Leica Byosystems Newcastle Ltd., UK) were identified after overnight incubation at 4°C. Sections were incubated with a secondary poly-HRP anti-mouse/rabbit IgG reagent (Bond Polimer Refine Detection; Leica Byosystems Newcastle Ltd., UK) against ER, PR, and Ki-67. The slides were developed with diaminobenzidine (DAB), counterstained with Mayer hematoxylin, dehydrated in ethanol and xylene, and finally mounted. Immunohistochemical staining was performed using the avidin-biotin complex method with antibodies direct against the following antigens: synaptophysin (1 : 100, Dako, Glostrup, Denmark), chromogranin A (1 : 700, Dako), neuron-specific enolase (1 : 200, Dako), carcinoembryonic antigens (CEA) (1 : 100, Dako), vimentin (1 : 700, Dako), and E-cadherin (1 : 60, Dako). P16 immunostain was performed using a monoclonal antibody to the p16^INK4A^ antigen (E6H4, 1 : 100 dilution; CINtec Histology, Heidelberg, Germany). All immunostains were performed using a Ventana Benchmark LT and XT automated immunostainers (Ventana Medical Systems, Tucson, AZ). Polymerase chain reaction (PCR) method was made to send the presence of HPV. DNA was extracted from the neoplastic tissue included in paraffin blocks. DNA amplification was made using a “Rotor-Gene Q” (Qiagen, Germany). DNA extraction from formalin-fixed and paraffin-embedded samples was performed using a Qiagen kit. Molecular analysis was performed by nested polymerase chain reaction (PCR) method using primer outer MY09/11 and primers inner GP5+/6+ for amplification L1 region using AB-Analitica Kit. Mucicarmine, PAS, and alcian blue were used to highlight the mucin content in the tumor signet-ring cells.

## 3. Results

**Figure 1:**
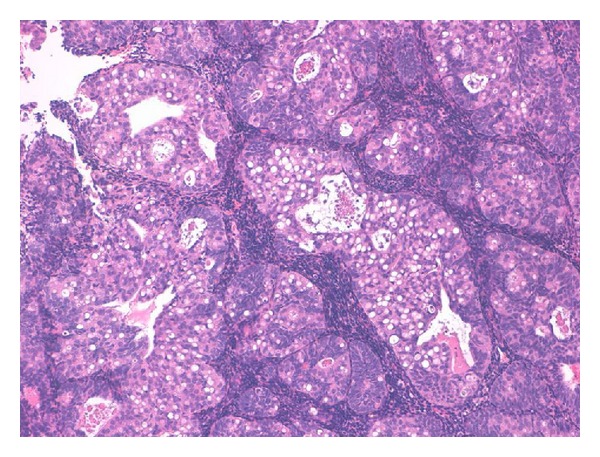
Endometrial curettage showed a neoplasm composed of glandular elements and solid areas with signet-ring cells (10x, hematoxylin and eosin).

**Figure 2:**
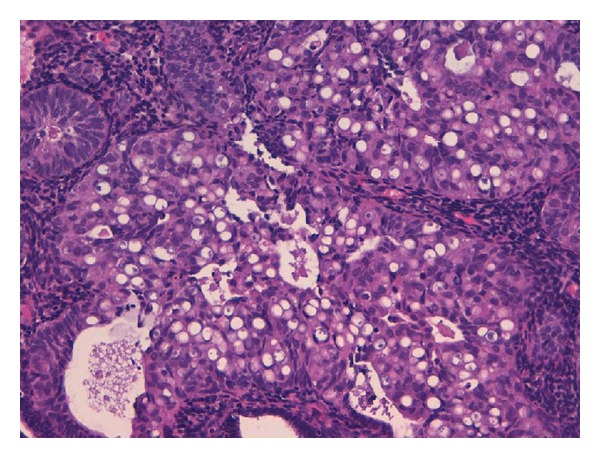
Signet-ring cells with vacuolated cytoplasm (20x, hematoxylin and eosin).

**Figure 3:**
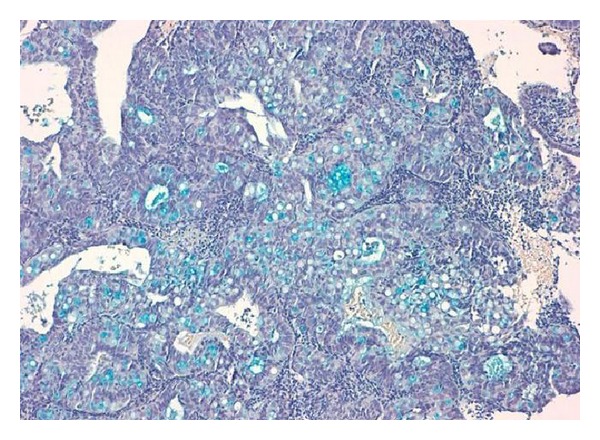
Alcian blue shows the presence of mucin in the signet-ring cells (10x alcian blue).

**Figure 4:**
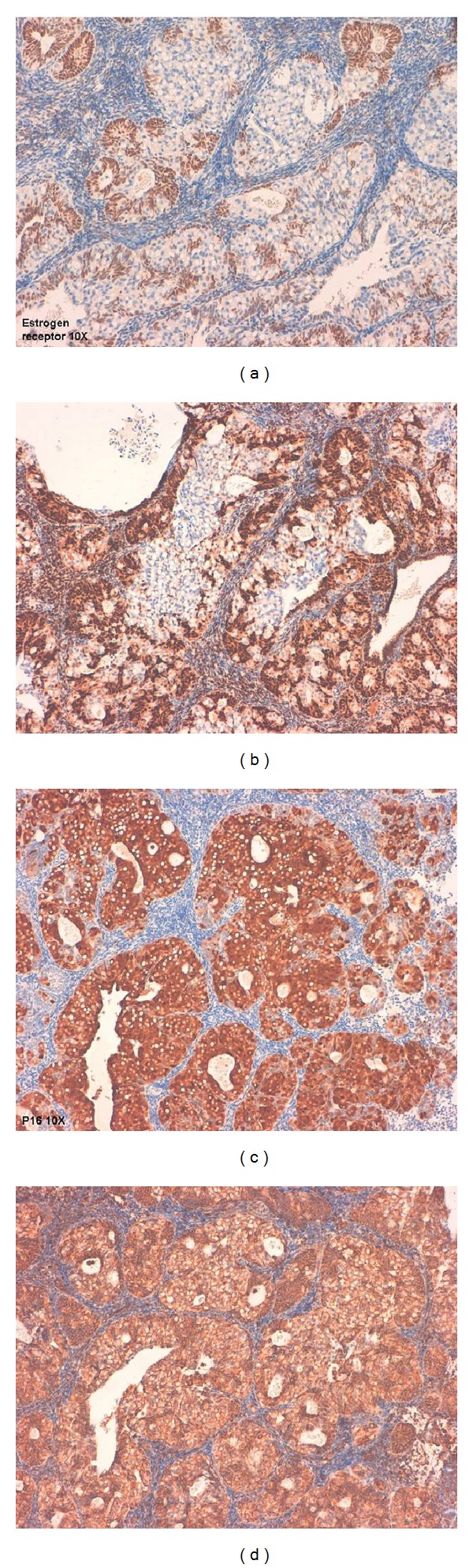
Estrogen Receptor (a) and Progesteron Receptor (b) immunostains are negative in the signet-ring cells component. P16 (c) and vimentin (d) immunostains are strongly positive in the signet-ring cell component (10x).

**Figure 5:**
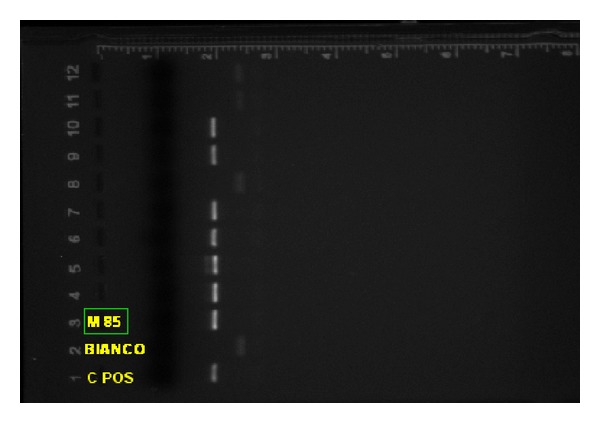
HPV positive view on agarose gel 2% (case identified as M-85).

**Figure 6:**
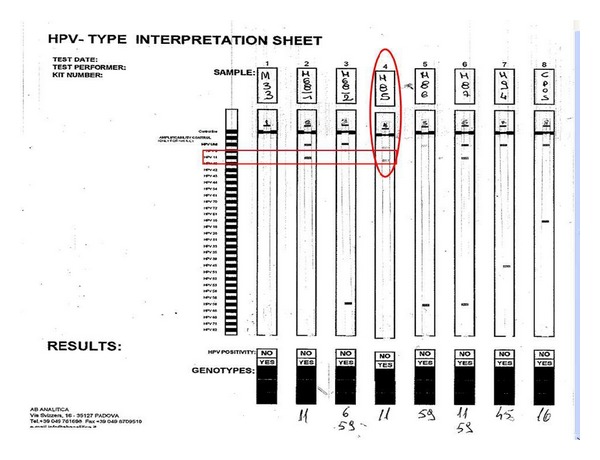
Genotyping case M-85 with HPV 11 positive.

FIGO grade 2 endometrioid adenocarcinoma (EA) of usual type with SRCs component was diagnosed in endometrial curettage (Figures 1 and 2). Foci of atypical endometrial hyperplasia were found. In the hysterectomy specimen, the moderately differentiated G2 endometrioid adenocarcinoma comprised 70% of the tumour, while 30% was represented by a SRCs component. The uterine neoplasm invaded less than one-half of the myometrium (FIGO stage I B). Isthmus and uterine cervix were free of disease. Special stains showed the presence of mucin in the SRCs component (Figure 3). ER and PR immunostains were negative in the SRCs tumoral component while they showed weak positivity in the endometrioid adenocarcinoma. The results of the additional immunostains were similar in the two components (Figure 4) (Table 2). Polymerase chain reaction method from paraffin-embedded tissue revealed the presence of human papilloma virus type 11 (Figures 5 and 6). The patient was alive and without evidence of recurrence 22 months after surgery.

## 4. Discussion

Among primary adenocarcinomas of the genital tract, SRCs tumours are generally rare. The World Health Organization (WHO) Classification [4] of the uterine cervix tumours describes SRC carcinoma as a rare variant in “pure form.” SRCs more commonly occur as a focal finding in poorly differentiated mucinous adenocarcinomas and adenosquamous carcinomas. WHO Classification [5] and the main gynaecologic pathology textbooks [6] do not report SRC carcinoma of the uterine corpus. To date four cases of endometrial adenocarcinoma with SRCs have been described in the literature. The tumour reported by Chebib et al. [2] was formed by two components and the percentage of each component was not specified. We think that the title of the paper is not accurate because the neoplasm is a mixed tumour composed of SRC carcinoma and endometrioid adenocarcinoma. Mooney et al. [1] report foci of atypical endometrial hyperplasia and FIGO grade I endometrioid carcinoma of the usual type in the endometrial curettage of a 51-year-old postmenopausal woman. Occasionally, vacuolated cytoplasm and SRCs were found. Mooney et al. [1] did not specify the percentage of the SRC component. We believe that the title of the paper is not accurate, so that the tumour would be defined as “endometrioid adenocarcinoma of the uterine corpus with SRCs component.” The title of the case described by Boyd et al. [3] is correct. The authors described a grade I EA in an endometrial polyp, with presence of small foci of solid tumour composed of SRCs. The subsequent hysterectomy did not reveal residual EA. It is evident that the histotype of the tumour is “endometrioid adenocarcinoma with SRCs.” The authors avoided the title “primary SRCs adenocarcinoma of the endometrium.” We believe that uterine neoplasm should be termed as “SRC adenocarcinoma of the endometrium” when the lesion is exclusively composed of SRCs. To date no case of true “primary SRC carcinoma of the endometrium” has been reported. The presence of SRCs cells in a carcinoma of the uterine corpus strongly raises the possibility of a metastasis. The most common extrauterine carcinomas that metastasize to or extend into the endometrium arise in the ovary, breast, or gastrointestinal tract, especially the colon. Therefore, abnormal uterine bleeding can be the first clinically apparent manifestation of disseminated disease [7, 8]. The myometrium is invaded by metastases more often than the endometrium. The extragenital malignancy that most frequently metastasizes to uterus is breast carcinoma; it is followed by primary gastrointestinal carcinomas, especially from stomach and colon. Metastatic breast carcinoma in the uterus is frequently characterized by polygonal cells or SRCs, often in a linear or single-file arrangement. An immunostain for gross cystic disease fluid protein-15 would help to identify the primary site of a breast metastasis. Metastatic gastrointestinal carcinomas are also characterized by SRCs or well-formed glandular structures. Immunohistochemical stains for CEA may be helpful in establishing whether the tumour is metastatic from the gastrointestinal tract. Generally, colonic primary tumours are diffusely positive for CEA while EAs are not. Also, endometrial carcinomas usually are positive for cytokeratin 7 and negative for cytokeratin 20, while metastatic colonic adenocarcinoma is usually strongly positive for cytokeratin 20 and negative for cytokeratin 7. HPV has emerged as one of the most important risk factors for human cancer and is recognized as an etiologic agent in virtually all cases of cervical cancers. Furthermore, HPV is also linked to other anogenital cancers as well as to a subset of head and neck cancers [9]. However, the relationship between HPV and other malignancies including upper genital tract, respiratory tract, digestive tract, and breast carcinomas is not clear [10, 11]. The role of HPV in endometrial carcinomas has been investigated giving contradictory results. The studies showed that the presence of HPV DNA in endometrial cancers differed in a range from 9% to 24%. In most cases HPVs were defined as high-risk type. HPV DNA, mostly 16 and 18 subtypes, was more intensively present in areas of squamous differentiation [12–16]. Karadayi et al. [17] believe that HPV does not play any role in the pathogenesis of endometrial carcinoma, since endometrium may not be a suitable host for HPV replication [17]. In our case the polymerase chain reaction (PCR) method revealed the presence of type 11 HPV. This finding is very surprising because the demonstration of the presence of HPV subtypes is usually used to support the cervical primitive origin of the adenocarcinoma. In our case the uterine cervix was free of tumour in the hysterectomy specimen. Immunohistochemical and molecular studies can help to distinguish endometrial from endocervical primary tumours. ER and PR immunostains were positive in EA, while they are negative in cervical adenocarcinomas. The combination of hormone receptors and HPV molecular detection appears to be very useful in this differential diagnosis. In our case ER and PR were negative in the SRC neoplastic component and weakly positive in the endometrioid adenocarcinoma. Preliminary data suggest that strong diffuse expression of p16^ink4^, which occurs in close to 100% of cervical squamous carcinomas and adenocarcinomas, is either absent or only patchy in endometrioid carcinomas [18]. p16^ink4^ is expressed strongly in lesions associated with intermediate- and high-risk HPV types, in contrast to low-risk HPV infection [19]. In selected head and neck squamous cell carcinomas, mainly from the oropharynx and sinonasal cavity, p16^ink4^ positivity correlates well with high-risk HPV infection. P16^ink4^ is not a reliable indicator of high-risk HPV infection in squamous cell carcinomas of the lung, Skin urinary bladder, and esophagus [20, 21].

## 5. Conclusion

In conclusion the expression of immunohistochemical markers was similar in the two components of the malignancy examined in the present study. The endometrial origin is documented by the histopathological examination of the hysterectomy specimen because uterine cervix and isthmus were free of disease.

## Figures and Tables

**Table 1:** Endometrial adenocarcinoma with signet-ring cells: review of the literature.

Case	Authors	Age/years	Diagnosis	Treatment	Stage	Followup
1	Mooney et al., 1997 [1]	65	SRC	Hysterectomy, B.S., pelvic and para-aortic L. partial O. abdominal, pelvic washing	NS	Free of disease 6 months after surgery

2	Chebib et al., 2010 [2]	51	Primary SRC	Hysterectomy, B.S., L. abdominal, pelvic washing	FIGO IVB	Death of metastatic disease 6 months after surgery

3	Boyd et al., 2010 [3]	46	Primary mucinous adenocarcinoma of the endometrium with signet-ring cells arising in adenomyosis	Subtotal hysterectomy	NS	NS

4	Boyd et al., 2010 [3]	59	Primary endometrioid adenocarcinoma of the endometrium with signet-ring cells	Hysterectomy	NS	NS

Our case		53	Primary endometrioid adenocarcinoma of the endometrium with signet-ring cells	Radical hysterectomy with B S L	FIGO stage IB	Free of disease 14 months after surgery

LLegend

SRCE: signet-ring cell carcinoma of the endometrium.

B.: bilateral.

S.: salpingo-oophorectomy.

L.: lymphadenectomy.

O.: omentectomy.

NS: not specified.

**Table 2:** Endometrioid carcinoma of the endometrium with signet-ring cells: immunohistochemical results in two tumoral components of our case.

Immunostaining	Endometrioid adenocarcinoma	Signet-ring cell carcinoma
CD56	Negative	Negative
Synaptophysin	Negative	Negative
CEA	Negative	Negative
Ki-67	5%	<2%
Chromogranin A	Negative	Negative
E-cadherin	Positive	Positive
HER-2	Negative	Negative
Estrogen receptor	Focally positive	Negative
Progesterone receptor	Weak positivity	Negative
Vimentin	Positive	Positive
p16	Positive	Positive
